# Host relatedness influences the composition of aphid microbiomes

**DOI:** 10.1111/1758-2229.12795

**Published:** 2019-10-20

**Authors:** Ailsa H. C. McLean, H. Charles J. Godfray, Jacintha Ellers, Lee M. Henry

**Affiliations:** ^1^ Department of Zoology University of Oxford Oxford UK; ^2^ Animal Ecology, Department of Ecological Science VU University Amsterdam Amsterdam The Netherlands

## Abstract

Animals are host to a community of microbes, collectively referred to as their microbiome, that can play a key role in their hosts' biology. The bacterial endosymbionts of insects have a particularly strong influence on their hosts, but despite their importance we still know little about the factors that influence the composition of insect microbial communities. Here, we ask: what is the relative importance of host relatedness and host ecology in structuring symbiont communities of diverse aphid species? We used next‐generation sequencing to compare the microbiomes of 46 aphid species with known host plant affiliations. We find that relatedness between aphid species is the key factor explaining the microbiome composition, with more closely related aphid species housing more similar bacterial communities. Endosymbionts dominate the microbial communities, and we find a novel bacterium in the genus *Sphingopyxis* that is associated with numerous aphid species feeding exclusively on trees. The influence of ecology was less pronounced than that of host relatedness. Our results suggest that co‐adaptation between insect species and their facultative symbionts is a more important determinant of symbiont species presence in aphids than shared ecology of hosts.

## Introduction

Animals are host to a large community of microbes, commonly referred to as their microbiome. Recent studies have shown that symbiotic microbes play significant roles in many aspects of their hosts' biology, including growth, metabolism, nutrition, digestion, protection and immunity (reviewed in Wernegreen, 2012; McFall‐Ngai *et al*., 2013; Russell *et al*., 2014). Although these studies have revealed the importance of microbes in host biology, we know little about the factors that determine the spectrum of microbial species living within particular eukaryotes and why microbes that are present in one host species may be absent in another.

Insects in particular carry an extraordinary diversity of symbiotic microbes that have played significant roles in their evolution. Many insects depend on obligate microbial symbionts (bacteria and fungi) to synthesize nutrients absent in their hosts' diets (Buchner, 1965; Douglas, 2009). In addition to these permanent symbioses, insects can harbour facultative symbionts that are not essential for their hosts' survival but may increase host fitness under certain ecological conditions. Facultative symbionts can protect their host from natural enemies, buffer against abiotic stresses such as heat shock and play a role in resource use (reviewed in Su *et al*., 2013; Hansen and Moran, 2014; Oliver *et al*., 2014); they are often transmitted maternally with high fidelity, although exchange between unrelated individuals also occurs (Russell *et al*., 2003). Although facultative symbionts can provide clear benefits to their host, the reasons for their absence in some individuals or populations within a species, and their mosaic distribution across species, are unclear.

Investigations into the factors influencing the structure of insect microbial communities have tended to find that host taxonomy has greater explanatory power than ecological factors such as diet or the environment (Chandler *et al*., 2011; Colman *et al*., 2012; Hawlena *et al*., 2012; Jones *et al*., 2013), although there are counter‐examples, such as in *Drosophila* species feeding on decaying mushrooms, in which four deeply diverged species have been found to host similar microbial communities (Martinson *et al*., 2017). Ecological factors, primarily the plant species insects feed on, are strongly correlated with the presence of certain species of facultative bacteria in at least two aphid species (Ferrari *et al*., 2008; Brady *et al*., 2014). Likewise, targeted studies searching for known symbionts among different pea aphid biotypes and across aphid species have shown that host ecology is correlated with the presence of particular facultative symbionts, with aphid phylogeny also playing a role, although of lesser importance (Henry *et al*., 2013; Gauthier *et al*., 2015; Henry *et al*., 2015).

Aphids (Aphidoidea) are a useful model for understanding the factors that influence insect microbiomes, because they possess a simple array of symbionts that occur across a broad taxonomic range of species and their ecologies (host–plant associations) are relatively well‐described. Previous studies of microbial diversity in aphids have typically used targeted sequencing techniques to search for previously described symbionts (e.g., Henry *et al*., 2013). A small number of studies have used next‐generation techniques to assess microbial communities; these have all been limited to a small number of aphid species and often a narrow taxonomic or ecological range (Jones *et al*., 2011; Brady and White, 2013; Russell *et al*., 2013; Gauthier *et al*., 2015; Jousselin *et al*., 2016; Fakhour *et al*., 2018). However, aphids provide an ideal opportunity to test the relative importance of phylogeny and ecology in determining the composition of insect microbiomes across a broad taxonomic range of species.

Here, we deep‐sequenced the bacterial 16S rRNA genes in 70 insect samples in order to compare the power of host phylogeny and ecology (food plant) in explaining the structure of the microbial community (both symbiont and non‐symbiont) in a phylogenetically diverse set of aphid species. We surveyed the microbial communities of 44 aphid species (plus two adelgids for comparison and control), selected from across the Aphidoidea, to explore four hypotheses. First, taxonomy matters: microbiomes will be more similar within aphid species than between aphid species. Second, another aspect of taxonomy: microbiome structure is correlated with observed genetic distance (p‐distance) between hosts. Third, ecology matters: different aphid species feeding on the same host plant will have more similar microbiomes than their phylogenetic position would suggest. Fourth, another aspect of ecology: different aphid species feeding on plants of the same growth form (herbs vs. trees) will have more similar microbial communities than predicted by their phylogenetic separation. Finally, our use of next‐generation sequencing techniques allows us also to discover whether previous studies based on targeted sequencing have missed important components of the aphid microbiota.

## Results and discussion

### *Relative abundance of bacteria*

The bacteria that were commonly found associated with aphids and adelgids (Operational Taxonomic Units (OTUs) present at frequencies >1% in any one sample) included (i) the obligate primary symbionts, *Buchnera* (aphids) and *Burkholdaria* (adelgids), (ii) known or suspected facultative symbionts on the basis of Oliver and colleagues (2010) and Zytynska and Weisser (2015), (iii) known or suspected insect and plant pathogens and (iv) species with unclear associations with aphids and their food plants (see Supporting Information Figs. S1 and S2 and Appendix S1). As expected, the primary symbionts of aphids and adelgids were always present and accounted for a median of 80.5% (min 8.9% to max 99.2%) of the sequences recovered from each host species. Bacteria belonging to the known facultative endosymbionts were the next most common and were found in 82.6% (38 of 46) of the insects sampled, making up, when present, a median of 6.7% (min 1.1% to max 87.5%) of the recovered sequences. In all but one case, next‐generation sequencing confirmed the presence of the well‐characterized symbionts identified by targeted polymerase chain reaction (PCR) using species‐specific primers (Henry *et al*., 2015). The exception was a single diverged *Serratia* lineage from a *Eulachnus* species that was only identified using the targeted sequencing approach.

Ten genera of known or suspected aphid facultative symbionts were found in the survey (Supporting Information Fig. S1 and Appendix S1). The most common facultative symbionts were placed in the genera *Hamiltonella* (found in 50% of species), *Serratia* (35%) and *Regiella* (22%). The symbiont *Serratia* can be very abundant in some aphids, equalling or exceeding *Buchnera* in sequences recovered (e.g., *Brachycaudus* sp. 1; see Supporting Information Fig. S1). Bacteria in the genus *Sphingopyxis* were harboured by 10 of the 44 (22%) aphid species, in some cases, at high relative abundance (Supporting Information Fig. S1). This is the first case of *Sphingopyxis* bacteria recorded from aphids, but this bacterium has been identified in other arthropods including two other Hemiptera (Zucchi *et al*., 2011; Pandey and Rajagopal, 2016) and ticks (Benson *et al*., 2004). The pattern of distribution and abundance in our samples is similar to other bacteria that are known to be symbionts, suggesting it too may be a facultative symbiont in aphids. However, experimental studies are needed to exclude other explanations such as it being an insect pathogen or a plant‐associated bacteria acquired through phloem feeding.

Unlike other insects that have diverse gut microbiota, containing many opportunistically acquired free‐living species, the relative abundance and diversity of non‐symbiotic bacteria in the aphid microbiome is low. Bacterial genera that contain known pathogens of insects (*Providencia* and *Pseudomonas*) and plants (*Erwinia* and *Xylella*) were detected in 21.7% (10 of 46) and 6.5% (3 of 46) of the insect individuals sampled, respectively, at low relative abundances (min‐median‐max: 1.1%–4.2%–21.8% and 1.3%–3.2%–7.3%; Supporting Information Figs. S2 and S3).

### *Distribution of facultative symbionts across the aphid phylogeny*

**Figure 1:**
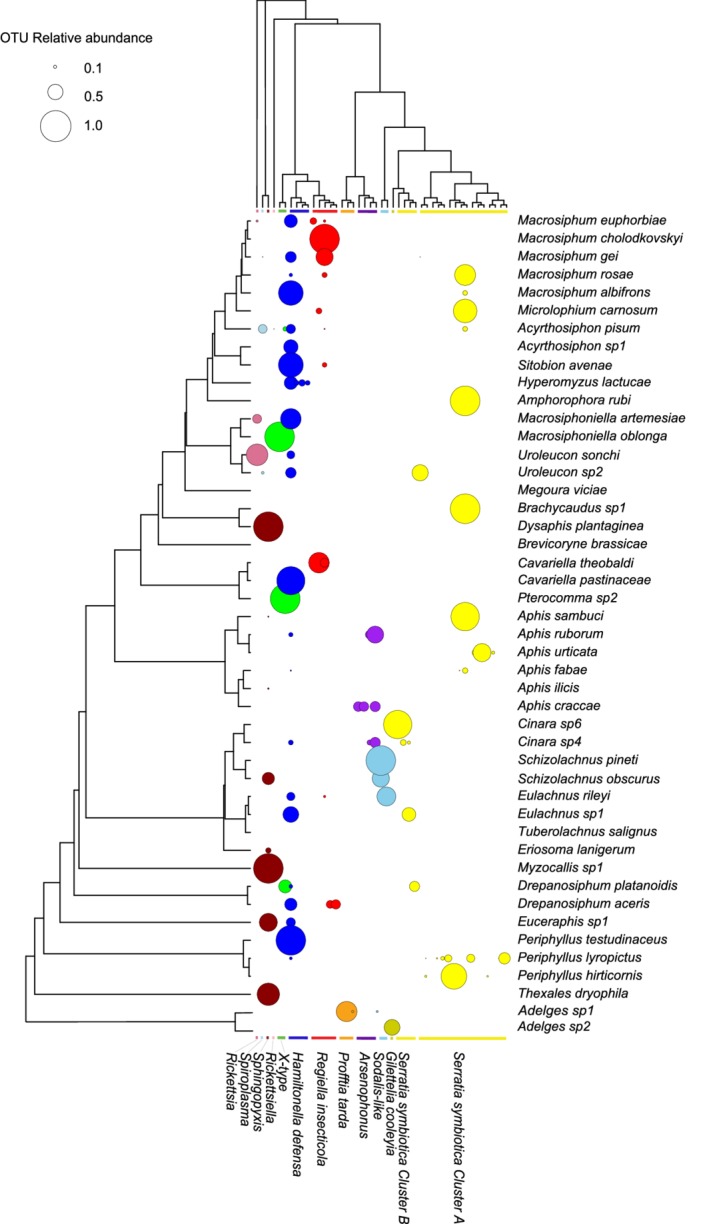
Interaction matrix for aphid species (*y*‐axis) and facultative symbiont lineages (*x*‐axis). Bacterial Maximum Likelihood phylogenies were bootstrapped 100 times. The aphid phylogeny was modified from a previously published tree (Henry *et al*., 2015) by pruning to only include the 46 species used in this study. The bacterial phylogeny is based on bacterial 16S sequences (V4 region) obtained in this study. Bubble size corresponds to the relative abundance of bacterial OTUs detected in each aphid species, excluding the primary symbionts (see Supporting Information Fig. S1 and Appendix S1 for actual abundances of all bacterial genera detected). The bacterial phylogeny is pruned to include only lineages that belong to known, or presumed, facultative symbionts of aphids. Colours correspond to the different genera of bacteria.

The distribution of facultative symbiont lineages across aphid species is displayed as an interaction matrix in Fig. 1 with the phylogenies of both host [based on the previously published phylogeny of Henry and colleagues (2015)] and symbiont. The bacterial phylogeny is pruned to include only lineages that cluster with known or putative genera of facultative symbionts (see Supporting Information Figs. S1 and S3), although we note that *Profftia* and *Gilettelia* in adelgids likely represent co‐obligate associations (von Dohlen *et al*., 2017). Similarly, the aphid genus *Cinara* has an apparently dynamic system of co‐obligate associations, with many species hosting *Serratia* as a co‐obligate symbiont (Lamelas *et al*., 2011), but with some examples in which other bacterial species including *Sodalis* and *Erwinia* play the co‐obligate role (Meseguer *et al*., 2017). The flexibility of bacterial lineages to move from facultative to obligate status inevitably complicates any attempt to make a strict division between the two categories.

Certain bacterial lineages (monophyletic groups within a symbiont species) infect many different aphid species, whereas others are restricted to a single aphid clade or species. This pattern is of course influenced by the choice of aphids we sampled. Facultative symbionts in the genera *Sphingopyxis*, *Sodalis* and *Serratia* ‘Cluster B’ are primarily found in aphids that are restricted to feeding on trees, mostly in the subfamily Lachninae. There are also cases where a symbiont lineage is restricted to a single aphid species (Fig. 1). For example, although the species *Hamiltonella defensa* is found infecting aphids across the entire phylogeny, two lineages are exclusively found infecting the aphid *Hyperomyzus lactucae*. Our results chime with previous reports such as that of Degnan and Moran (2008), who found that a specialized clade of *H*. *defensa* was present in all individuals within a monophyletic section of the aphid genus *Uroleucon*. The occurrence of these lineages within a single aphid species may indicate that the bacteria are no longer horizontally transferred to other aphid species and have formed a specialized relationship with the host, although further work is needed to test this hypothesis.

### *Community structure of aphid microbiomes*

**Figure 2:**
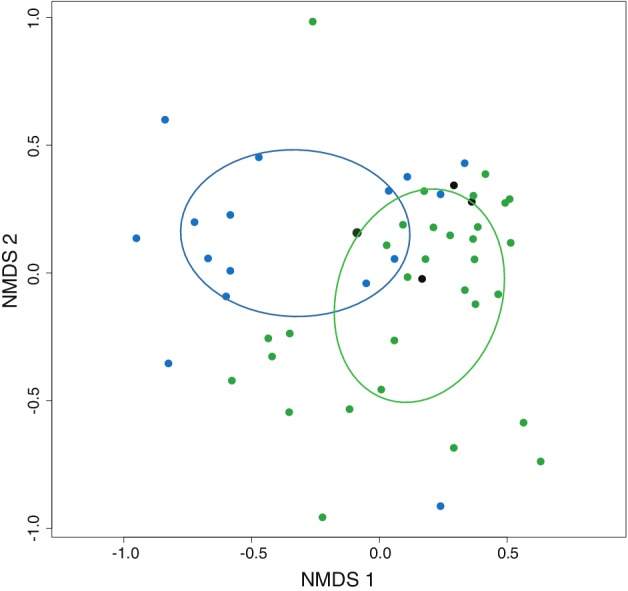
Non‐metric multidimensional scaling biplot of the similarity in bacterial communities harboured by aphids. NMDS ordination was performed on the relative abundance of bacterial OTUs in each sample using Bray–Curtis distances, with two axes specified. Coloured dots represent aphid species that feed exclusively on trees (blue) and herbs (green) or those that obligately alternate between trees and herbs each year (black). The distance between dots reflects the similarity in the bacterial communities between samples, with closer dots having more similar communities. Coloured ellipses represent 95% confidence internals of sample centroids that highlight the similarity of the microbiomes associated with aphids feeding on trees versus those on herbs.

We used NMDS ordination to visualize microbiome community structure across the aphid individuals we sampled. Aphids from the same species tend to be positioned near each other with the exception of individuals from *Aphis fabae* and *Acyrthosiphon pisum* (Supporting Information Fig. S4). These two aphid species are known to have a complex population structure made up of different biotypes (Peccoud *et al*., 2009). Tree‐ and herb‐feeding aphids tend to cluster together with host‐alternating species intermediate (Fig. 2). Different species of aphid feeding on the same plant tend not to cluster together (Supporting Information Fig. S4).

We used permutation tests of the Bray–Curtis similarity index to assess microbiome similarity and to test the different hypotheses. Taxonomic distance was the most important correlate of microbiome composition that we identified. For comparisons of both the facultative symbiont community and the non‐symbiont community, bacterial communities were more similar within aphid species, even when feeding on different host plants, than between aphid species (Hypothesis 1; Table 1, A1).

**Table 1:** Analysis of similarity between the microbial communities harboured by: (A) intra versus interspecific aphid species, (B) aphid species with different degrees of relatedness and (C) for aphids that share similar versus different ecologies.

A.				
Hypothesis	Mean Bray–Curtis index	Mean Bray–Curtis index	Difference	*p* value
1	Intraspecific (*n* = 19 comparisons)	Interspecific (*n* = 309 comparisons)		
All OTUs	0.56	0.86	−0.29	<0.0001
Symbiont OTUs	0.60	0.84	−0.23	<0.0001
Non‐symbiont OTUs	0.71	0.93	−0.22	<0.0001

B.			
Hypothesis	Intercept	Slope	*p* value
2a	Aphid relatedness regression (same plant; *n* = 36 comparisons)		
All OTUs	0.81	1.11	<0.0001
Symbiont OTUs	0.83	0.80	<0.0001
Non‐symbiont OTUs	0.89	0.57	<0.0001
2b	Aphid relatedness regression (different plant; *n* = 1535 comparisons)		
All OTUs	0.83	0.85	<0.0001
Symbiont OTUs	0.83	0.80	<0.0001
Non‐symbiont OTUs	0.88	0.73	<0.0001
2c	Difference in slope H2a versus H2b		
All OTUs	–	–	0.36
Symbiont OTUs	–	–	0.44
Non‐symbiont OTUs	–	–	0.46

C.				
Hypothesis	Mean Bray–Curtis index	Mean Bray–Curtis index	Difference	*p* value
3	Same plant (*n* = 36)	Different plants (*n* = 1535)		
All OTUs	0.92	0.92	0.003	0.55
Symbiont OTUs	0.94	0.95	−0.04	0.10
Non‐symbiont OTUs	0.91	0.91	0.004	0.55
4	Herb versus Herb (*n* = 494) Tree versus Tree (*n* = 186)	Herb versus Tree (*n* = 640)		
All OTUs	0.88	0.94	−0.05	<0.0001
Symbiont OTUs	0.87	0.93	−0.05	<0.0001
Non‐symbiont OTUs	0.93	0.96	−0.03	<0.0001

Hypotheses above were tested using permutation tests of the Bray–Curtis similarity index. Sample sizes (numbers of comparisons made) are given in brackets; details of contrasts are given in Appendix S2.

Hypotheses.

**1** – Microbiomes are more similar within aphid species than between aphid species.

**2 –** Microbiome structure correlates with genetic distance between aphid species.

**2a** – More closely related aphid species have more similar microbiomes (aphids on the same food plants).

**2b** – More closely related aphid species have more similar microbiomes (aphids on different food plants).

**2c** – The difference between related aphid species will be greater when they feed on different plants.

**3** – Aphid species on the same plants have more similar microbiomes than aphid species feeding on different plants.

**4** – Aphids that live on plants of the same growth form (trees vs. herbs) harbour more similar bacterial communities than those living on plants of different growth form.

**Figure 3:**
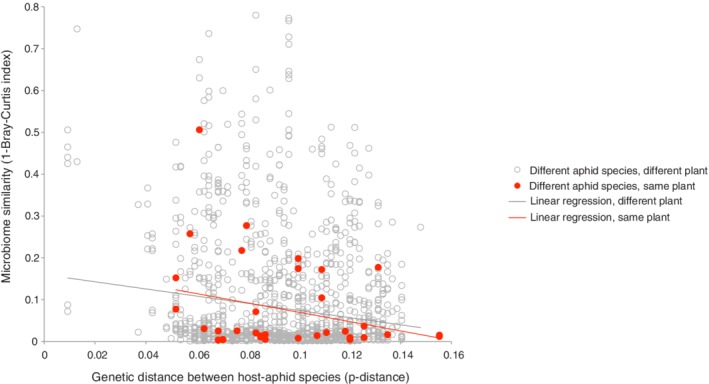
Relationship between the genetic distance of aphid species (*x*‐axis) and the similarity of the microbial communities they host (*y*‐axis), excluding primary symbionts. Community similarity is measured using the Bray–Curtis dissimilarity index, with values of zero representing identical communities. Red circles are comparisons of two different aphid species feeding on the same species of plants, whereas open grey circles are pairwise comparisons of different aphid species on different plant species, with accompanying trend lines.

We found that microbiome similarity was correlated with the genetic distance (p‐distance) between hosts (Hypothesis 2; Fig. 3; Table 1, A2a‐c), with more closely related host species harbouring more similar microbial communities. We calculated the regression separately for aphids feeding on the same plant species (Table 1, A2a) and on different plant species (Table 1, A2b) but found no difference in the slope (Table 1, A2c). Aphids of different species feeding on the same host plant did not have more similar communities than aphids feeding on different host plants (Hypothesis 3) for either the symbiont or non‐symbiont fractions of the bacterial community (Table 1, B3). Aphids feeding on the same host plant growth form (tree or herb) were found to have more similar bacterial communities (Hypothesis 4) when comparing tree–tree and herb–herb distances using permutation tests (Table 1, B4). This was true for both symbiont and non‐symbiont bacteria. However, because different aphid taxa feed on trees and herbs, phylogenetic and ecological determinants are confounded and statistical methods to disentangle them at the community level are not available.

## Conclusions

Overall, our results are consistent with host taxonomy being the key factor determining the composition of microbial communities in aphids. This is somewhat surprising given that previous studies of two individual aphid species (pea aphid, *A*. *pisum*, and cowpea aphid, *Aphis craccivora*) have found that aphid populations adapted to different plant species tend to harbour particular facultative symbiont species, suggesting plant ecology is important in explaining the presence of symbionts in aphids (Frantz *et al*., 2009; Ferrari *et al*., 2012; Brady *et al*., 2014; Peccoud *et al*., 2015). However, both of these aphid species comprise genetically differentiated populations (biotypes) that are adapted to feed on different plant species and it is possible that host genetics may also play a role in explaining the presence of symbionts *within* these aphid species. Recent experimental evidence supports host genetics playing a role in symbiont transfer among aphid biotypes (Parker *et al*., 2017; Niepoth *et al*., 2018).

Why should more closely related aphids harbour similar bacterial communities? This is likely to be due to a combination of vertical transmission of facultative symbionts through aphid matrilines and a higher likelihood of horizontal transfer between more closely related aphid species. Aphid symbionts are more likely to establish an infection in a new host, and produce a stable association, when artificially transferred between more closely related host species (Łukasik *et al*., 2015), suggesting host‐mediated selection and co‐adaptation between insects and their facultative microbes can impact the symbiont component of the aphid microbiome. Other studies comparing microbiomes across species have also demonstrated that host taxonomy has an important influence on the microbial community structure in insects (Chandler *et al*., 2011; Colman *et al*., 2012; Hawlena *et al*., 2012; Jones *et al*., 2013). It has been suggested that the facultative symbionts of aphids and other insects could function as a ‘horizontal gene pool’, analogous to bacterial plasmids, allowing exchange of beneficial genetic material between host lineages (Jaenike, 2012; Henry *et al*., 2013); our results indicate that host phylogeny is likely to shape patterns of symbiont exchange within the horizontal gene pool.

In summary, by characterizing the bacterial microbiome of a broad taxonomic range of aphid species, we have shown that host relatedness is the main correlate of microbiome structure in aphids. The microbial community is dominated by obligate and facultative symbiont species, the majority (but possibly not all) of which have already been identified from work on a small number of model aphid species. The importance of symbionts and other microbial associates to their hosts' biology, including their capacity to cause damage as pests and transmit diseases, is becoming increasingly apparent. Placing the microbiome of individual species within the context of broader host community and phylogeny is an important step towards understanding the evolution and function of microbiome communities.

## Experimental procedures

Our study material consisted of 70 samples, each comprising a pool of 6–10 (mean: 7.7) individuals of the same insect species collected from a single plant species (42 plants in total) and from a minimum of three study sites. Genomic DNA was extracted from individual specimens using QIAGEN DNeasy Blood and Tissue kits and the V4 region of the bacterial 16S gene amplified using PCR (Caporaso *et al*., 2012). Individuals were then combined into pools for deep‐sequencing using the Illumina MiSeq2000 platform. Following filtering and quality control, sequences were clustered into OTUs at 97% identity. Rare OTUs (<1% relative abundance) and OTUs identified as resulting from sequencing or PCR errors were removed. The remaining OTUs were assigned to a taxonomic group using the ‘Greengenes’ database v 13.8 (McDonald *et al*., 2012) and cross‐referenced with GenBank using BLASTn. Number of OTUs for each sample, and each bacterial genus, are shown in Appendix S1.

We compared the microbial communities of different aphid species that share the same host plant species (*N* = 14 plant species with 2–4 aphid species per plant), and in polyphagous aphids collected from multiple species of plants (*N* = 11 aphid species collected from 2–3 plant species), using pairwise Bray–Curtis dissimilarity indexes (a full list of comparisons is given in Appendix S2). Differences in microbiome composition were analysed for statistical significance using permutation tests on the Bray–Curtis indexes. For this analysis, OTUs belonging to the obligate symbionts were removed. We subsequently divided the community into ‘OTUs belonging to known facultative symbionts’ and ‘OTUs belonging to non‐symbionts’ and re‐ran the analysis to determine if there are differences between the two classes of microbial associate. Full details of all experimental procedures are given in the Supporting Information.

## Supporting information


**Appendix S1:** Total and absolute OTUs of each bacterial genera in each sample

**Appendix S2:** Aphid and plant contrasts included in each analysis (NAs indicate contrasts not included)

**Appendix S3:** Supporting information

**Figure S1** Relative abundance of all bacterial genera in 44 species of aphids and 2 species of adelgids. Abundance is based on the proportion of OTUs belonging to each bacterial lineage clustered at 97% similarity of 16S sequence data.

**Figure S2** Relative abundance of different categories of bacteria in 44 species of aphids and 2 species of adelgids. Bacteria lineages are assigned to categories based on their known function in aphids. Abundance is based on the proportion of OTUs belonging to each bacterial lineage clustered at 97% similarity of 16S sequence data.

**Figure S3** Evolutionary relationships of the most common bacterial lineages (>1% relative abundance) harboured by the aphids and adelgids in this study. Maximum likelihood bacterial phylogeny is based on the 16S sequence data from this study (blue font) and published sequence data (black font) retrieved from GenBank. Nodes with >50 bootstrapping support are identified on the phylogeny. The tips of the phylogeny are labelled with the bacteria lineages OTU identity that are >97% similar to published sequences in GenBank. For bacterial lineages only found in a single host species, the aphid/adelgid name is indicated in brackets. Lineages that are <97% similarity to published 16S reads are highlighted in red. Bacterial lineages that cluster with known facultative symbionts are highlighted with vertical lines on the right of the phylogeny.

**Figure S4** Non‐metric Multidimensional Scaling biplots of the similarity in bacterial communities harboured by aphids. NMDS ordination was performed on the relative abundance of bacterial OTUs in each sample using Bray‐Curtis distances, with 2 axes specified (see SOM for details). (A) Samples of the same aphid species collected from different plant species. Aphid species are connected with solid lines of the same colour and labelled with the aphid species name. (B) Samples of different aphid species that feed on the same plant species. Aphid species are connected with hatched lines of the same colour. Coloured dots represent aphid species that feed exclusively on trees (blue), herbs (green), or those that obligately alternate between trees and herbs each year (black). The distance between dots reflects the similarity in the bacterial communities between samples, with closer dots having more similar communities.

## Data Availability

The sequence data have been submitted to the NCBI Sequence Read Archive, BioProject accession no. PRJNA573918.
