# Supplementary material for: Host relatedness influences the composition of aphid microbiomes
Source: Environ Microbiol Rep. 2019 Oct 20;11(6):808–16. doi: 10.1111/1758-2229.12795 (PMC6900097; doi:10.1111/1758-2229.12795)
Supplement: Supplementary file 3 — Appendix S3: Supporting information [file EMI4-11-808-s003.docx]

**Experimental procedures**

*Processing of samples*

Our study material consisted of 70 samples. Each sample comprised a pool of 6-10 (mean: 7.7) individuals of the same insect species (44 aphid and 2 adelgid species), collected from a single plant species (42 plants in total) and from a minimum of three study sites. Within each locality, collections were separated by at least 10m to reduce the probability of re-sampling the same aphid clone. On collection, all aphids were stored in 90% ethanol prior to DNA extraction. To test for the importance of food-plants on aphid microbiomes some aphid species were collected from multiple plant species and some plant species were sampled for multiple aphid species. The aphid species used in this study were chosen from a larger set of samples to provide a set of species contrasts that i) vary in their degree of phylogenetic relatedness, and ii) share similar versus different ecologies (food-plants). A list of the aphid species and the plants from which they were collected is presented in Appendix 1 of the Supplementary Online Materials.

All aphid specimens were surface-sterilised using 100% ethanol before use. Aphids used in this study had been previously identified to species using morphological examination and by DNA barcoding (Henry *et al*., 2015). Genomic DNA was extracted from individual specimens using QIAGEN DNeasy Blood and Tissue kits and we amplified an approximately 700 bp DNA fragment of the cytochrome c oxidase I (COI) mitochondrial gene, which was sequenced in the forward direction using the same primers. Molecular identification to species was achieved by comparing the COI sequence to the on-line databases BOLD (www.barcodinglife.org) and GenBank using BLAST.

*Bacterial 16S rRNA sequencing*

We sampled the bacterial community of the 61 samples by deep-sequencing the universal bacterial 16S rRNA gene. We PCR-amplified the V4 region of the bacteria 16S gene from the genomic DNA of individual aphids following standard protocols (Caporaso *et al.,* 2012), and then combined individuals prior to deep-sequencing the pooled amplicons using the Illumina MiSeq2000 platform at the Centre for Genomic Research, Institute of Integrative Biology, University of Liverpool. Sequencing used 4 lanes (2 ‘pools’ in each direction) and a single-indexing workflow, with primers (F515/R806) suitable for multiplex sequencing with Illumina technology (Caporaso *et al.,* 2011). Standard 16S rRNA analysis techniques, including sequence trimming, filtering, chimera removal, Operational Taxonomic Units (OTUs) selection, and taxonomic assignments of reads were performed using the QIIME version 1.8.0 bioinformatics toolkit (Caporaso *et al.,* 2010). After filtering and quality control there were a total of 21 Million reads across all samples. Reads were not normalized across samples, but depth of coverage per sample was deemed sufficiently high to capture OTU diversity within each sample. Sequences were clustered into OTUs at 97% identity using the furthest neighbor algorithm, which generated 136554 OTUs (many of which will be the result of sequencing or PCR errors). After removal of rare OTUs (those <1% relative abundance) and OTUs generated by sequencing or PCR errors, 146 dominant OTUs remained present at >1% abundance in any one sample. The single most abundant 16S sequence was used as a representative for each of the OTUs clustered at 97% similarity, henceforth referred to as a bacteria lineage. We used the 'Greengenes' databases version 13.8 (McDonald *et al.,* 2012) to assign each of the representative OTU sequences to a taxonomic group. The total number of OTUs for each sample, and the absolute number of OTUs for each bacterial genus (at >1% relative abundance) are presented for each sample in Appendix 1 (SOM).

*Phylogenetic analysis*

To produce Figure 1, we estimated the phylogenetic relationship of the most prevalent (>1% relative abundance) bacterial lineages in our samples using the 16S rRNA sequence data (only a sub-sample of sequences from the primary symbiont *Buchnera aphidicola* are included) (Figure S3). A Maximum Likelihood phylogeny was generated for the bacterial OTUs using the on-line PhyML server (Guindon *et al.,* 2010), and the best fitting models of evolution was estimated using the Aikake Information Criterion (AIC). The bacterial phylogeny was bootstrapped 100 times and rooted to a 16S sequence from *Thermus thermophiles*, which is basal to all of the bacterial species found in this study (Wu *et al.,* 2009). The bacterial phylogeny was rendered ultrametric for character mapping using the ‘chronopl’ function in the APE package in R. The aphid phylogeny was modified from a previously published tree (Henry et al 2015) by pruning to only include the 46 species used in this study using the ‘drop.tip’ function in R. For each aphid species, the relative abundance of each facultative bacterial lineage was mapped onto the tips of the aphid phylogeny, and their phylogenetic distribution was visualized using the APE package version 4.1 (Paradis *et al.,* 2004) in R v.3.1.1 (R Development Core Team 2013).

The names at the tips on the bacterial phylogenetic tree (Figure S3) are based on the taxonomic names for each OTU assigned by the Greengenes database, which were confirmed by comparing to published records on Genbank using BLAST. Only sequences with >97% similarities are labelled with the genus or species names. Those OTUs with >3% difference from any known species record are noted on the phylogeny.

Bacterial lineages were labelled as being known “symbionts”, “pathogens” or “plant-related” if they were >97% similar to sequences from studies that have used experimental, genomic or morphological techniques to classify the bacteria as being either harmful, or intracellular facultative or obligate symbionts in aphids and adelgids, or those that were plant symbionts. Where information on the biology of the bacteria was not available, lineages were designated as unknown.

*Estimates of microbiome community structure*

We estimated the importance of aphid species and their host plant (species and plant growth form, e.g. trees versus herbs) to microbiome composition by comparing the microbial communities of different aphid species that share the same host plant species (*N*=14 plant species with 2–4 aphid species per plant), and in polyphagous aphids collected from multiple species of plants (*N*=11 aphid species collected from 2–3 plant species) (Appendix 1). The similarity of the bacterial communities associated with each sample was estimated using pairwise Bray-Curtis dissimilarity indexes, which is a measure to quantify the compositional dissimilarity of microbes between samples based on counts of OTU units clustered at 97%, using the VEGAN package in R v.3.1.1 (R Development Core Team 2013, Oksanen *et al.,* 2018). The similarity of bacterial communities in each sample was compared to all other samples. For this analysis, we removed OTUs belonging to the obligate symbionts that are present in all species of aphids (*Buchnera aphidicola*) and adelgids (*Burkholderia* spp.) to focus on the presence and absence of facultative microbes.

The similarity of the bacterial communities associated with different aphids species was initially assessed and visualized using Non-metric Multidimensional Scaling (NMDS), which is a means of visualizing the level of similarity between samples. NMDS ordination was performed on the relative abundance of bacterial OTUs in each sample using Bray-Curtis distances, with 2 axes specified, using the metaMDS function in VEGAN. Coloured 95% confidence ellipses in Figure 2 are based on the standard error of the mean centroid of treatments and were produced using the ‘ordiellipse’ function in VEGAN.

*Statistical analysis*

The composition of the microbial community an insect carries may be influenced by several factors, including its phylogenetic position and its ecology (the latter represented here by food-plant species and growth form). Our specific hypotheses are given above (see Introduction). Significant differences in the composition of microbiomes associated with different groups of aphids were analysed using permutation tests on the Bray-Curtis indexes. To test hypothesis 1, we asked whether the microbiomes of the same aphid species feeding on different plants (*N*=11 aphid species) were more similar than between-species comparisons using these same 11 aphid species. To test hypothesis 2, we compared the microbiomes of all unique aphid species-plant combinations and asked if more closely related aphid species have more similar microbiomes. We used as a measure of aphid phylogenetic relatedness the proportion of CO1 nucleotide sites at which the two aphid species differ (*p*-distance), and excluded intraspecific comparisons where the same aphid species was collected on multiple host plants. To address hypothesis 3, we asked if different aphid species that feed on the same plant species (*N*=14 plant species on which two or more aphid species fed) have more similar microbiomes than different aphid species that feed on different plant species. To test hypothesis 4, we asked if comparisons made between aphid species that fed on the same plant growth form (herbs or trees) have more similar microbial communities than comparisons between pairs of species where one feeds on herbs and the other on trees. Aphid species that alternate host plants from trees to herbs were excluded from this analysis. A full account of the aphid species comparisons is presented in Appendix 2 (SOM).

We tested the above hypotheses using all the bacterial OTUs, excluding primary symbionts. We then divided the community into “OTUs belonging to known facultative symbionts” and “OTUs belonging to non-symbionts” and re-ran the analysis to determine if the factors structuring populations of facultative endosymbionts differ from non-symbiotic bacteria (including pathogens, free-living bacteria etc.).

*Experimental Procedures References*

Caporaso, J. G., J. Kuczynski, J. Stombaugh, K. Bittinger, F. D. Bushman, E. K. Costello, N. Fierer, A. G. Peña, J. K. Goodrich, J. I. Gordon, G. A. Huttley, S. T. Kelley, D. Knights, J. E. Koenig, R. E. Ley, C. A. Lozupone, D. McDonald, B. D. Muegge, M. Pirrung, J. Reeder, J. R. Sevinsky, P. J. Turnbaugh, W. A. Walters, J. Widmann, T. Yatsunenko, J. Zaneveld and R. Knight (2010). "QIIME allows analysis of high-throughput community sequencing data." Nature Methods 7: 335.

Caporaso, J. G., C. L. Lauber, W. A. Walters, D. Berg-Lyons, J. Huntley, N. Fierer, S. M. Owens, J. Betley, L. Fraser, M. Bauer, N. Gormley, J. A. Gilbert, G. Smith and R. Knight (2012). "Ultra-high-throughput microbial community analysis on the Illumina HiSeq and MiSeq platforms." ISME J 6: 1621.

Caporaso, J. G., C. L. Lauber, W. A. Walters, D. Berg-Lyons, C. A. Lozupone, P. J. Turnbaugh, N. Fierer and R. Knight (2011). "Global patterns of 16S rRNA diversity at a depth of millions of sequences per sample." Proc Natl Acad Sci USA 108(Supplement 1): 4516-4522.

Guindon, S., J.-F. Dufayard, V. Lefort, M. Anisimova, W. Hordijk and O. Gascuel (2010). "New Algorithms and Methods to Estimate Maximum-Likelihood Phylogenies: Assessing the Performance of PhyML 3.0." Syst Biol 59(3): 307-321.

McDonald, D., M. N. Price, J. Goodrich, E. P. Nawrocki, T. Z. DeSantis, A. Probst, G. L. Andersen, R. Knight and P. Hugenholtz (2012). "An improved Greengenes taxonomy with explicit ranks for ecological and evolutionary analyses of bacteria and archaea." ISME J 6: 610.

Oksanen, J., F. G. Blanchet, M. Friendly, R. Kindt, P. Legendre, D. McGlinn, P. R. Minchin, R. B. O'Hara, E. Szoecs and H. Wagner (2018). vegan: Community Ecology Package. R package version 2.4-6. <https://CRAN.R-project.org/package=vegan>.

Paradis, E., J. Claude and K. Strimmer (2004). "APE: Analyses of Phylogenetics and Evolution in R language." 20(2): 289-290.

R Development Core Team (2013). R: a language and environment for statistical computing. <http://www.R-project.org/>.

Wu, D., P. Hugenholtz, K. Mavromatis, R. Pukall, E. Dalin, N. N. Ivanova, V. Kunin, L. Goodwin, M. Wu, B. J. Tindall, S. D. Hooper, A. Pati, A. Lykidis, S. Spring, I. J. Anderson, P. D’haeseleer, A. Zemla, M. Singer, A. Lapidus, M. Nolan, A. Copeland, C. Han, F. Chen, J.-F. Cheng, S. Lucas, C. Kerfeld, E. Lang, S. Gronow, P. Chain, D. Bruce, E. M. Rubin, N. C. Kyrpides, H.-P. Klenk and J. A. Eisen (2009). "A phylogeny-driven genomic encyclopaedia of Bacteria and Archaea." Nature 462: 1056.
