# Supplementary figures and images for: Host relatedness influences the composition of aphid microbiomes
Source: Environ Microbiol Rep. 2019 Oct 20;11(6):808–16. doi: 10.1111/1758-2229.12795 (PMC6900097; doi:10.1111/1758-2229.12795)

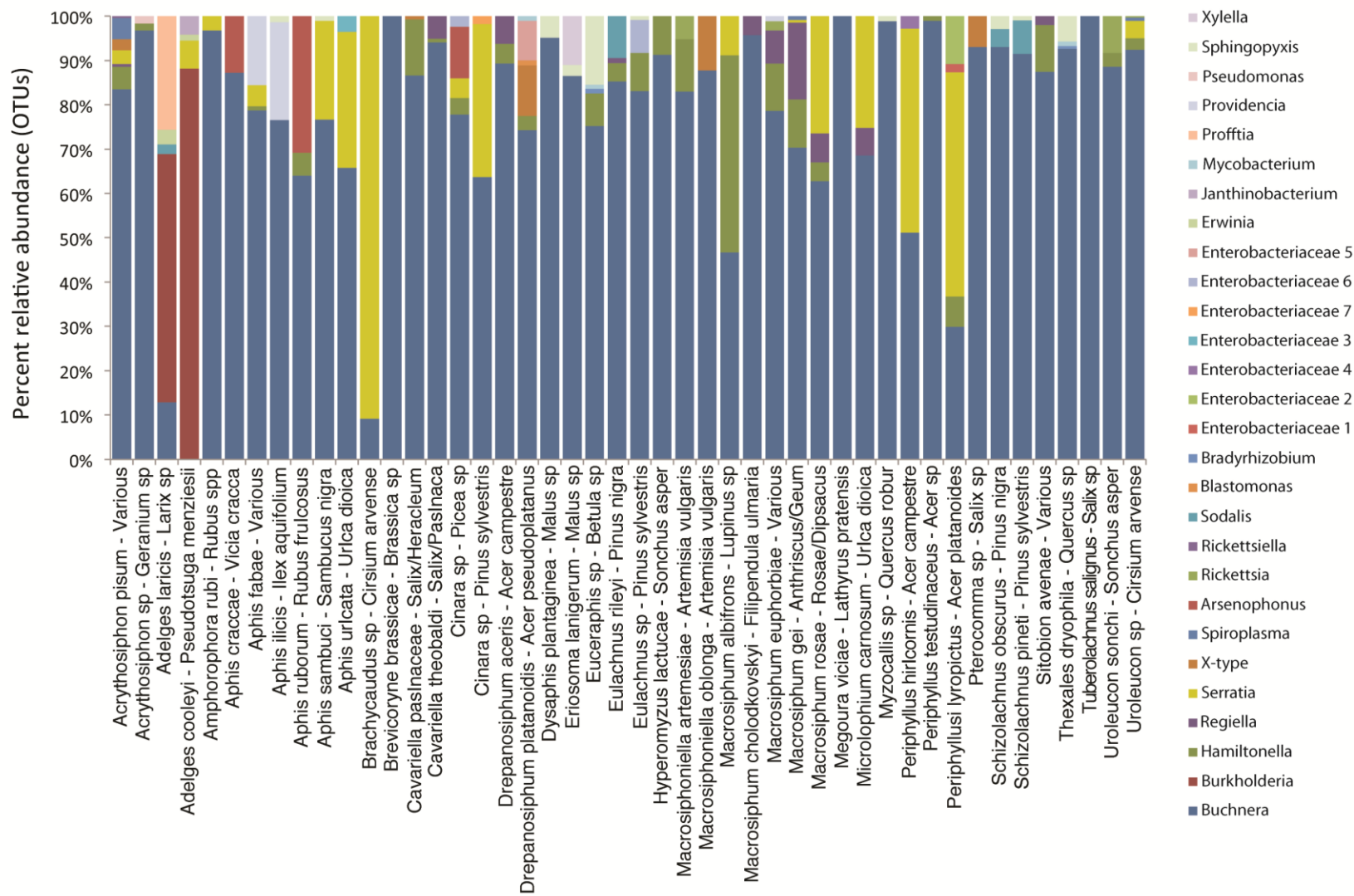

Supplement: Supplementary file 4 — Figure S1 Relative abundance of all bacterial genera in 44 species of aphids and 2 species of adelgids. Abundance is based on the proportion of OTUs belonging to each bacterial lineage clustered at 97% similarity of 16S sequence data. [file EMI4-11-808-s002.pdf]

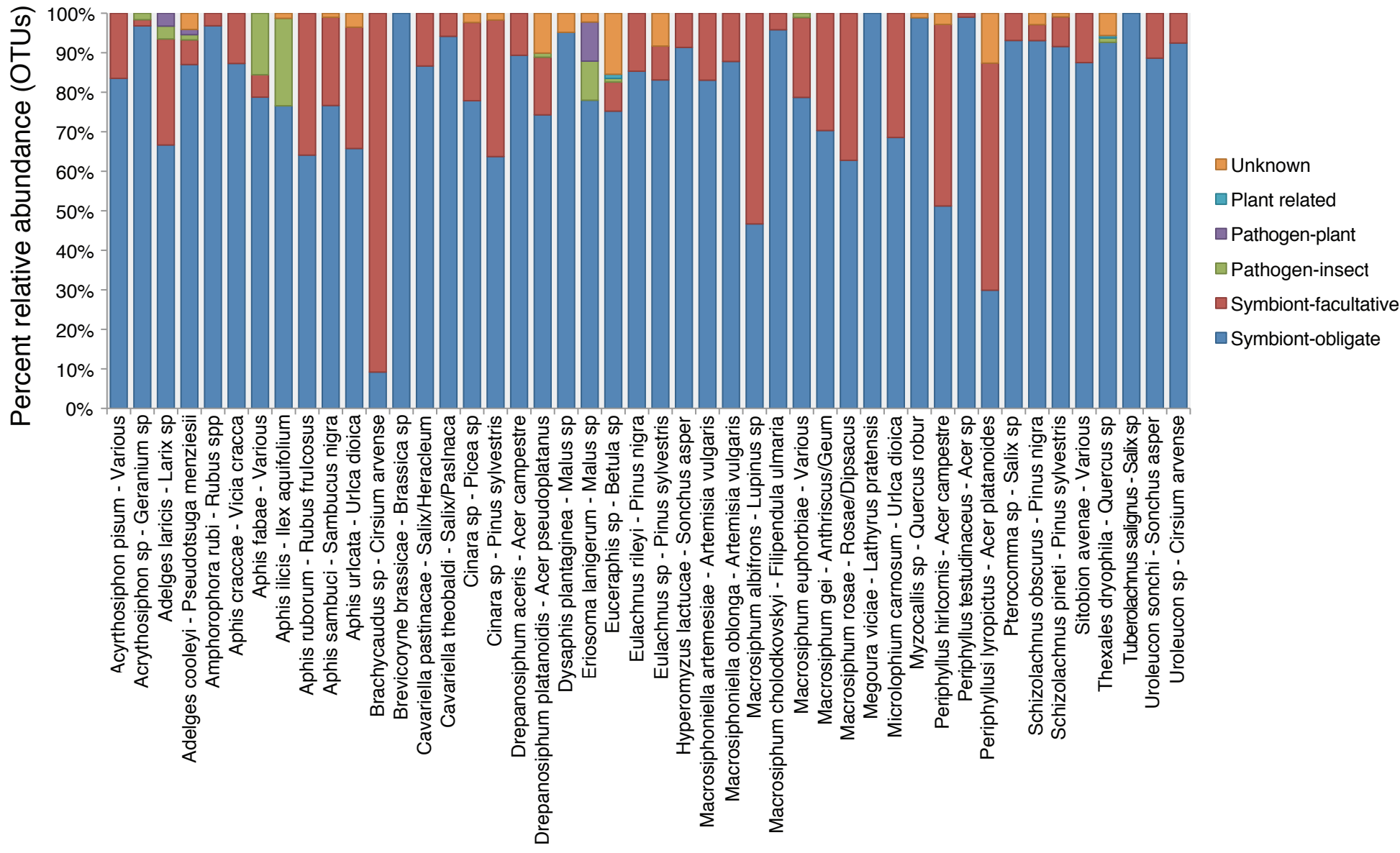

Supplement: Supplementary file 5 — Figure S2 Relative abundance of different categories of bacteria in 44 species of aphids and 2 species of adelgids. Bacteria lineages are assigned to categories based on their known function in aphids. Abundance is based on the proportion of OTUs belonging to each bacterial lineage clustered at 97% similarity of 16S sequence data. [file EMI4-11-808-s005.pdf]

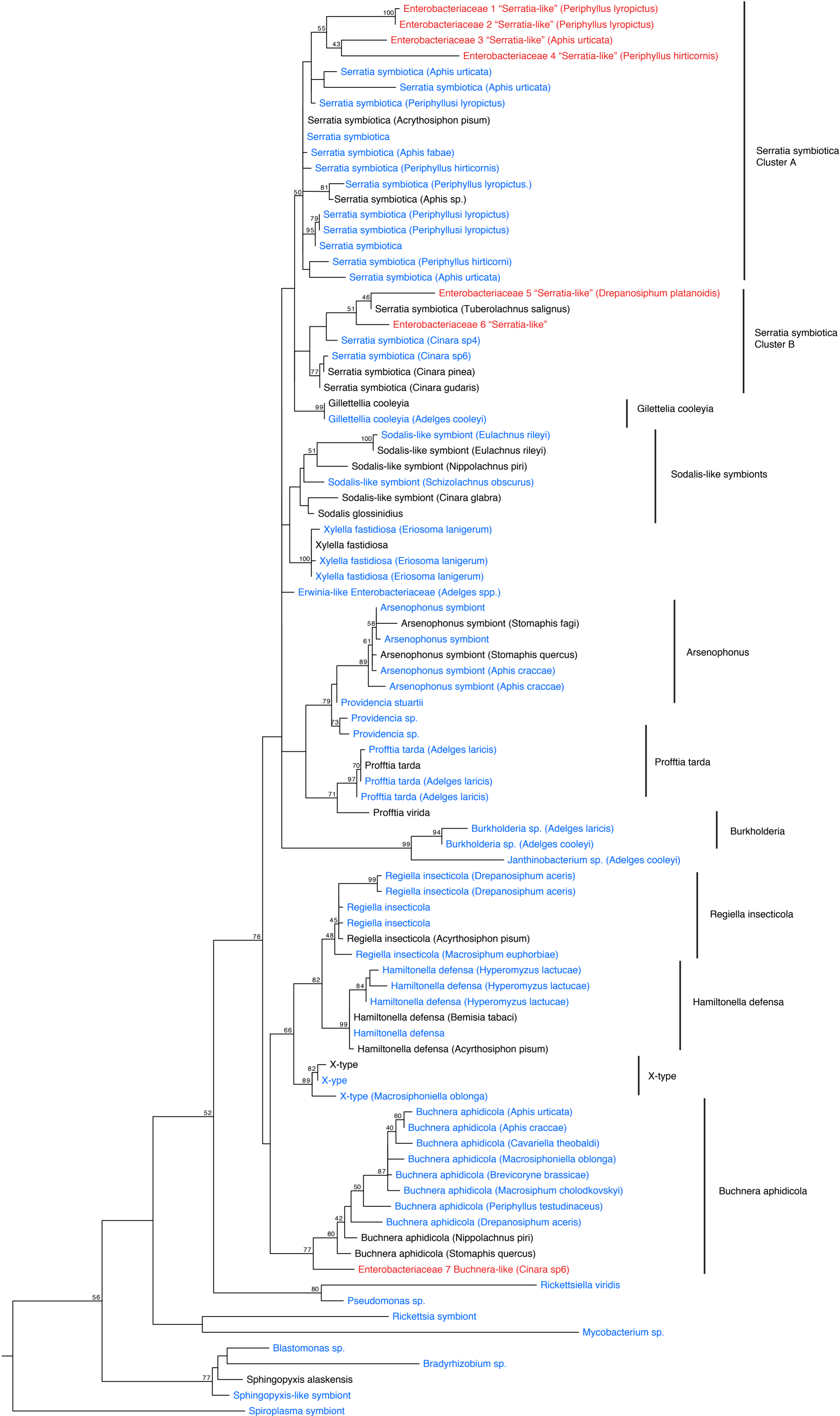

Supplement: Supplementary file 6 — Figure S3 Evolutionary relationships of the most common bacterial lineages (>1% relative abundance) harboured by the aphids and adelgids in this study. Maximum likelihood bacterial phylogeny is based on the 16S sequence data from this study (blue font) and published sequence data (black font) retrieved from GenBank. Nodes with >50 bootstrapping support are identified on the phylogeny. The tips of the phylogeny are labelled with the bacteria lineages OTU identity that are >97% similar to published sequences in GenBank. For bacterial lineages only found in a single host species, the aphid/adelgid name is indicated in brackets. Lineages that are <97% similarity to published 16S reads are highlighted in red. Bacterial lineages that cluster with known facultative symbionts are highlighted with vertical lines on the right of the phylogeny. [file EMI4-11-808-s006.pdf]

A.

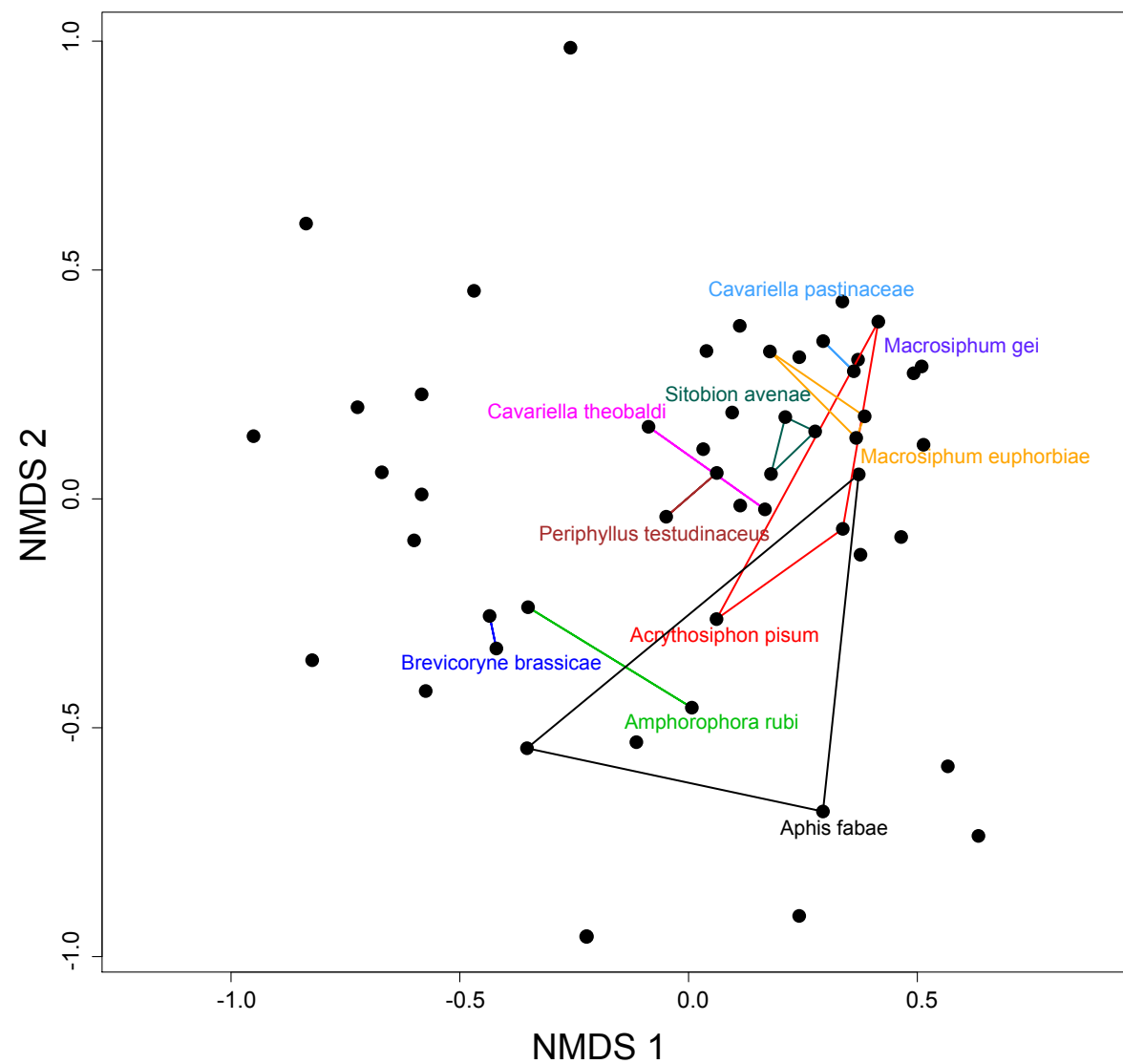

B.

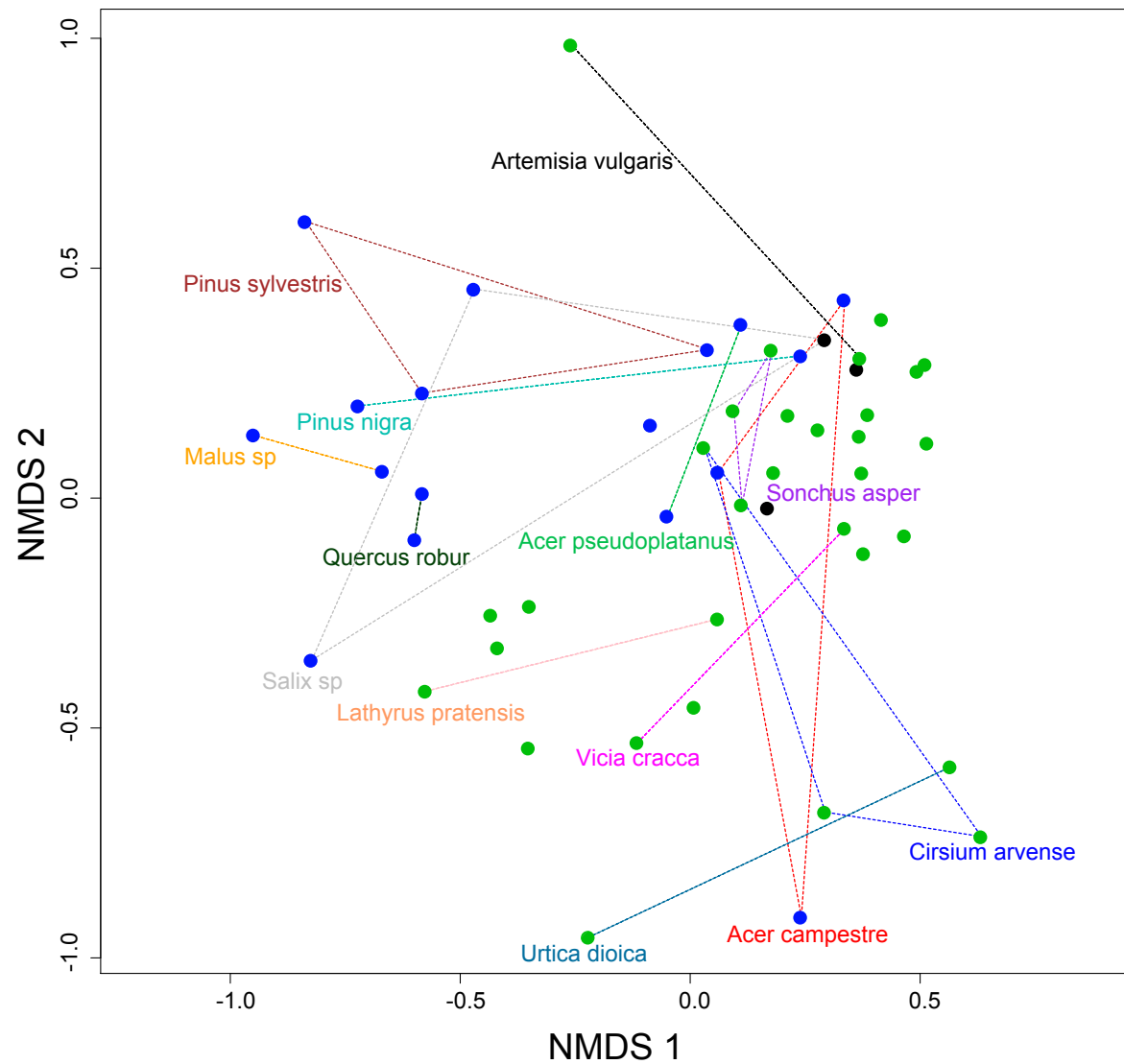

Supplement: Supplementary file 7 — Figure S4 Non‐metric Multidimensional Scaling biplots of the similarity in bacterial communities harboured by aphids. NMDS ordination was performed on the relative abundance of bacterial OTUs in each sample using Bray‐Curtis distances, with 2 axes specified (see SOM for details). (A) Samples of the same aphid species collected from different plant species. Aphid species are connected with solid lines of the same colour and labelled with the aphid species name. (B) Samples of different aphid species that feed on the same plant species. Aphid species are connected with hatched lines of the same colour. Coloured dots represent aphid species that feed exclusively on trees (blue), herbs (green), or those that obligately alternate between trees and herbs each year (black). The distance between dots reflects the similarity in the bacterial communities between samples, with closer dots having more similar communities. [file EMI4-11-808-s007.pdf]
